# 

**Published:** 1858-06

**Authors:** 


No. I. JUNE—1858. Vol. XIV.

### BUFFALO MEDICAL JOURNAL

AND

### Monthly Review

OF

### MEDICAL AND SURGICAL SCIENCE.

AUSTIN FLINT, Jr., M. D.,

EDITOR.

BUFFALO, N. Y.

E. R. JEWETT, PUBLISHER.

Commercial Advertiser Buildings.

Price $2.50, payable in advance, or $3.00 if not paid till the end of the year.

